# Sitagliptin Alleviates Radiation-Induced Intestinal Injury by Activating NRF2-Antioxidant Axis, Mitigating NLRP3 Inf--lammasome Activation, and Reversing Gut Microbiota Disorder

**DOI:** 10.1155/2022/2586305

**Published:** 2022-05-17

**Authors:** Shanshan Huang, Yongbiao Huang, Wanling Lin, Lei Wang, Yang Yang, Piao Li, Lei Xiao, Yuan Chen, Qian Chu, Xianglin Yuan

**Affiliations:** ^1^Department of Oncology, Tongji Hospital, Huazhong University of Science and Technology, Wuhan 430030, China; ^2^Department of Organ Transplantation, Tongji Hospital, Huazhong University of Science and Technology, Wuhan 430030, China; ^3^Division of Cardiology, Department of Internal Medicine, Tongji Hospital, Huazhong University of Science and Technology, Wuhan 430030, China

## Abstract

Radiation-induced intestinal injury is a common and critical complication of radiotherapy for pelvic or abdominal tumors, with limited therapeutic strategies and effectiveness. Sitagliptin, a dipeptidyl peptidase IV (DPP4) inhibitor, has previously been reported to alleviate total body irradiation- (TBI-) induced damage of hematopoietic system in mice, but its effect on radiation-induced intestinal injury remains unclear. In this study, we confirmed that Sitagliptin could not only protect mice from death and weight loss caused by whole abdominal irradiation (WAI) but also improve the morphological structure of intestine and the regeneration ability of enterocytes. In addition, Sitagliptin significantly inhibited the production of radiation-induced proinflammatory cytokines and reduced the number of apoptotic intestinal epithelial cells and *γ*-H2AX expression. *In vitro*, we demonstrated that Sitagliptin protected HIEC-6 cells from ionizing radiation, resulting in increased cell viability and reduced DNA damage. Mechanistically, the radiation protection of Sitagliptin might be related to the upregulation of NRF2 level and the decrease of NLRP3 inflammasome activity. Importantly, Sitagliptin significantly restored radiation-induced changes in bacterial composition. In conclusion, our results suggested that Sitagliptin could reduce WAI-induced intestinal injury in mice, which may provide novel therapeutic strategy for radiation-induced intestinal injury.

## 1. Introduction

Radiotherapy is one of the common treatments for malignant tumors. However, radiation-induced normal tissues damage greatly limits the clinical application of radiotherapy [1]. The small intestine is particularly sensitive to ionizing radiation, which can disrupt the intestinal mucosal barrier and lead to acute gastrointestinal (GI) syndrome or even life-threatening symptoms [2–4]. Therefore, there is an urgent need to identify an effective drug to promote the regeneration of intestinal epithelial cells and reduce radiation-induced gastrointestinal toxicity.

Sitagliptin, an FDA-approved oral hypoglycemic agent, is one of the dipeptidyl peptidase IV (DPP-4) inhibitors. It inhibits the degradation of glucagon-like peptide-1 (GLP-1) and glucose-dependent insulinotropic polypeptide, resulting in the biological effect of inhibiting glucagon secretion and promoting insulin secretion [5]. As research progresses, Sitagliptin has been proven to play a therapeutic role in a series of diseases such as atherosclerosis, ischemia-reperfusion injury, acute lung injury, and Alzheimer's disease [6–9]. Its protective effect seems to be related to its anti-inflammatory, antioxidant, and antiapoptotic properties. The latest study suggests that Sitagliptin may be a potential radiation protective agent, which can alleviate 7.5 Gy total body irradiation- (TBI-) induced death and reduce the radiation-induced damage of hematopoietic system in mice [10]. However, the effectiveness of Sitagliptin in preventing or mitigating radiation-induced intestinal injury remains unknown.

In the present study, we investigated whether Sitagliptin made a protective effect in radiation-induced intestinal injury and its underlying mechanisms. Our results demonstrated that the administration of Sitagliptin had therapeutic effects on radiation-induced intestinal damage, which protected mice from whole abdominal irradiation- (WAI-) induced death, inhibited radiation-induced inflammation, apoptosis, and DNA damage, and promoted the regeneration of intestinal epithelial cells in irradiated mice. In addition, we revealed that its protection was associated with the upregulation of NRF2 expression, inhibition of NLRP3 inflammasome activation, and regulation of gut microbiota disorder.

## 2. Materials and Methods

### 2.1. Animals and Chemicals

Male C57BL/6 mice (6-8 weeks, 20-22 g) were bought from Jiangsu GemPharmatech Co., Ltd. (Nanjing, China), and bred in standard laboratory conditions in the Tongji Hospital Laboratory Animal Centre. All animal experiments were permitted by the Animal Ethics Committee of Tongji Hospital, Tongji Medical College, Huazhong University of Science and Technology. Sitagliptin was purchased from MedChemExpress (New Jersey, USA).

### 2.2. Irradiation (IR) and Sitagliptin Treatment in Intestinal Injury Model

A clinical linear accelerator (6Mv X-rays, Elekta Precise, Stockholm, Sweden) at a dose rate of 0.98 Gy per minute was used for all experiments; the exact does was measured using a three-dimensional (3D) water scanning system (IBA blue phantom, Germany). Mice were randomized into four groups: control (Con), Sitagliptin, WAI, and WAI+Sitagliptin. For the intestinal experiments, all mice were anaesthetized with 1.5% sodium pentobarbital during IR, the WAI and WAI+Sitagliptin groups received a single dose of 12.5 Gy WAI (X-rays, 6Mv), and the Con and Sitagliptin groups were sham-irradiated. All mice were administered with Sitagliptin (20 mg/kg) or vehicle (PBS) via oral gavage 1 hour before WAI/sham-irradiated and followed once a day for 3 days after WAI. The 20 mg/kg dose and the dosing schedule were based on the previous literatures [11–14]. For the *in vitro* experiments, HIEC-6 cells were treated complete medium with or without Sitagliptin 1 hour before IR and then irradiated with a single dose of 6 Gy X-rays or sham-irradiated.

### 2.3. Histological Analysis and Immunohistochemistry (IHC)

The isolated small intestines were fixed in 4% paraformaldehyde, embedded in paraffin, and then cut into 4 *μ*m sections for hematoxylin-eosin (HE) staining, periodic acid-Schiff (PAS) staining, and IHC staining in accordance with the manufacturer's instructions. The primary antibodies used for IHC staining included anti-Lgr5 antibody (1 : 2500, Boster, BM4244), anti-Lysozyme antibody (1 : 1000, Boster, BA0092), anti-Villin antibody (1 : 5000, Proteintech, Cat No. 66096-1-Ig), anti-Ki67 antibody (1 : 100, Abcam, ab15580), and anti-*γ*-H2AX antibody (1 : 100, CST, #9718). All sections were observed and imaged under a microscope (Leica, Germany), and positive cells were quantified by two blinded pathologists.

### 2.4. Immunofluorescence (IF)

The small intestines were processed as for IHC, slides were blocked with 10% normal donkey serum and incubated with the primary antibodies anti-NRF2 antibody (1 : 200, CST, #12721), anti-*γ*-H2AX antibody (1 : 400, CST, #9718), and anti-C-caspase3 (1 : 200, CST, #9664) overnight at 4°C, followed by fluorescent secondary antibody at 37°C for 1 h. Finally, the slides were stained with DAPI (Servicebio, China) and photographed using a fluorescence microscope (Leica, Germany).

### 2.5. TUNEL Assay

The sections were stained using the TUNEL assay staining kit (Roche, Germany) according to the manufacturer's protocol and previous study [15].

### 2.6. Quantitative Real-Time PCR (qRT-PCR)

According to the manufacturer's protocol, total RNA was extracted from the small intestine tissues or HIEC-6 cells using Trizol (TAKARA, Japan) and then reverse transcribed to cDNA using PrimeScript™ RT reagent (TAKARA, Japan), and the qRT-PCR was performed using SYBR Green Real-Time PCR assay kit (TAKARA, Japan) with the 7900HT RT-PCR system (Applied Biosystems, USA). All primers used are shown in Table S1.

### 2.7. Malondialdehyde (MDA) Measurement

The MDA content of small intestine tissues was measured using the corresponding kits (Nanjing Jiancheng Bioengineering Institute, China) according to the manufacturer's protocol.

### 2.8. Cell Culture

The human enterocyte cell line HIEC-6 and luciferase positive murine colon cancer cell line MC38-luc were cultured in RPMI-1640 medium (Hyclone, USA) containing 10% fetal bovine serum at 37°C with 5% CO_2_.

### 2.9. Cell Viability

HIEC-6 cells (5 × 10^3^ cells per well) were plated into 96-well plates and incubated for 24 h, and then, different concentrations of Sitagliptin were added into medium. After another 24 h treatment, CCK-8 reagents (MedChemExpress, USA) were added to assess cell viability using absorbance detected by a microplate reader (BioTek, USA) at 450 nm wavelength.

### 2.10. Western Blot Analysis

Total cellular protein was extracted using RIPA buffer (Servicebio, China) as described previously. The extracted proteins were separated by SDS-PAGE and transferred to polyvinylidene fluoride membranes. Membranes were blocked with 5% BSA and incubated with following primary antibodies. Then, the membranes were washed and incubated with secondary antibodies and analyzed using the G:BOX Chemi X system (Syngene, UK).

### 2.11. Transplantation Tumor and Irradiation Experiment

The MC38-luc cells (1 × 10^6^) were subcutaneously injected into the right flank of C57BL/6 mice. Seven days after tumor transplantation (the volume of the tumors reached 250 mm^3^ approximately) [16, 17], the mice were divided into six groups randomly: Con group, Sitagliptin (20 mg/kg) group, Sitagliptin (100 mg/kg) group, IR group, IR+Sitagliptin (20 mg/kg) group, and IR+Sitagliptin (100 mg/kg) group. All mice were anaesthetized with 1.5% sodium pentobarbital during IR and treated with PBS or different concentrations of Sitagliptin or a single dose of 8 Gy local IR or combination of IR with Sitagliptin. Sitagliptin was administered by oral gavage one hour before local IR and followed once a day until the maximum tumor volume exceeded 2000 mm^3^. The tumor size and mouse weight were measured every two days. According to the formula tumor volume = length × width^2^/2, tumor volume was calculated. All tumor tissues were collected for immunohistochemistry stained with Ki-67.

### 2.12. *In Vivo* Optical Imaging Analysis

Mice were intraperitoneally injected with D-fluorescein and anesthetized with 1.5% pentobarbital sodium. Optical imaging was detected by Lago X system (Spectral Instruments Imaging).

### 2.13. Intestine Microbiome Composition Analysis

Fresh feces were collected and stored at liquid nitrogen used for 16S ribosomal RNA (rRNA) sequencing. According to the standard protocol, the 16S rRNA sequencing was performed using an Illumina HiSeq platform (Novogene Technology Co., Ltd., China) to assess the intestine microbiome composition. All sequencing data were analyzed as described previously [18].

### 2.14. Statistical Analysis

All statistical analyses were performed by GraphPad Prism 8.0 (San Diego, USA), and the data are presented as mean ± standard error of the mean. Differences were compared using unpaired Student's *t*-tests for two groups or one-way ANOVA for multiple groups. Survival analysis was performed by the Kaplan–Meier method and compared with the log-rank test. *P* < 0.05 was considered statistically significant. ^∗^*P* < 0.05, ^∗∗^*P* < 0.01, and ^∗∗∗^*P* < 0.001. Graphs show mean ± SD.

## 3. Results

### 3.1. Sitagliptin Improves Survival and Attenuates Radiation-Induced Intestinal Injury

**Figure 1:**
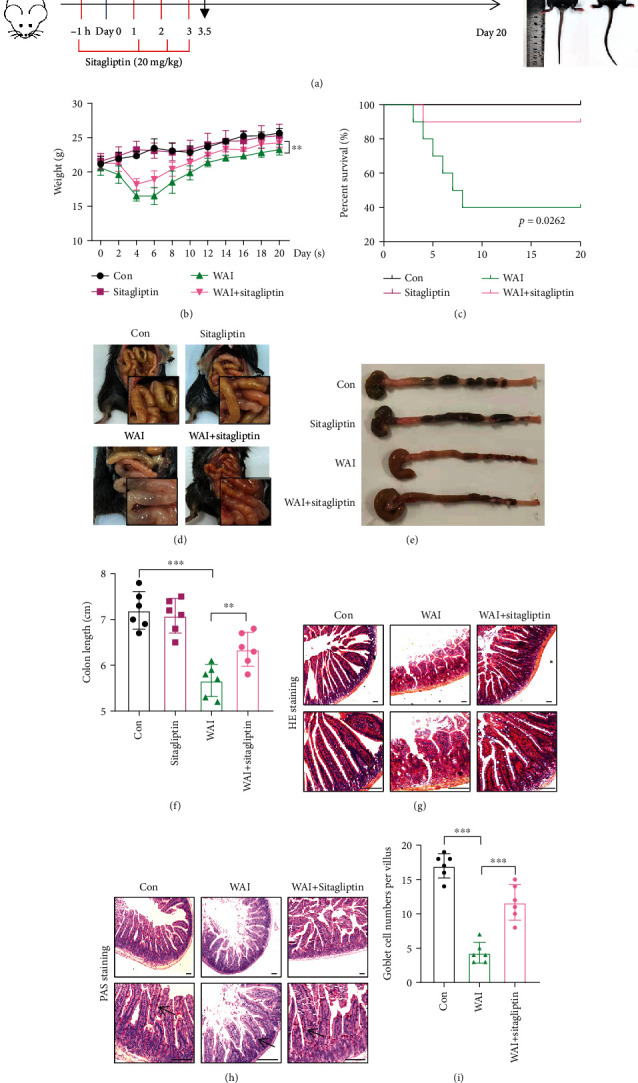
Sitagliptin alleviates intestinal damage in mice with WAI. (a) Operational diagram of the animal experiment with WAI. (b, c) Body weight and survival analysis of mice in the Con (*n* = 6), Sitagliptin (*n* = 6), WAI (*n* = 10), and WAI+Sitagliptin (*n* = 10) groups. (d) Macroscopic appearance of the intestine in each group. (e, f) Colon length in each group. (g) H&E staining of the small intestine from indicated groups. Scale bars, 100 *μ*m. (h) PAS staining analysis of the small intestine from indicated groups. (i) Quantification of goblet cells based on PAS staining. Scale bars, 100 *μ*m, *n* = 6.

To explore the impact of Sitagliptin in radiation-induced intestinal injury, we administrated Sitagliptin orally 1 hour before the C57BL/6 mice received 12.5 Gy whole abdominal irradiation (WAI), and Sitagliptin was then given once a day for another three days (Figure 1(a)). Obviously, oral administration of Sitagliptin significantly slowed weight loss and improved survival in the irradiated group (Figures 1(b) and 1(c)).

Based on previous studies, intestinal tissues were collected 3.5 days after WAI in mice [14]. As expected, WAI resulted in reduced intestinal contents and severe hemorrhagic and congestive changes in the small intestine. Sitagliptin treatment alleviated the symptoms and maintained intestinal contents in irradiated mice (Figure 1(d)). Moreover, Sitagliptin significantly attenuated the colon length shortening of irradiated mice (Figures 1(e) and 1(f)). Histological analysis of intestinal sections by H&E staining showed that the small intestinal villi of the irradiated mice were degenerated and shortened, and the crypt was damaged, while Sitagliptin treatment significantly increased the length of villus and crypt in mice after WAI (Figure 1(g)). Goblet cells, which play a critical role in barrier function, were shown to be significantly reduced in mice at 3.5 days after WAI by PAS staining. On the contrary, Sitagliptin could markedly increase the goblet cells of the small intestine in irradiated mice (Figures 1(h) and 1(i)). Collectively, our results support the mind that treatment with Sitagliptin could alleviate WAI-induced intestinal injury in mice.

### 3.2. Sitagliptin Inhibits Intestinal Inflammation and Promotes the Regeneration of Intestinal Epithelial Cells in Irradiated Mice

**Figure 2:**
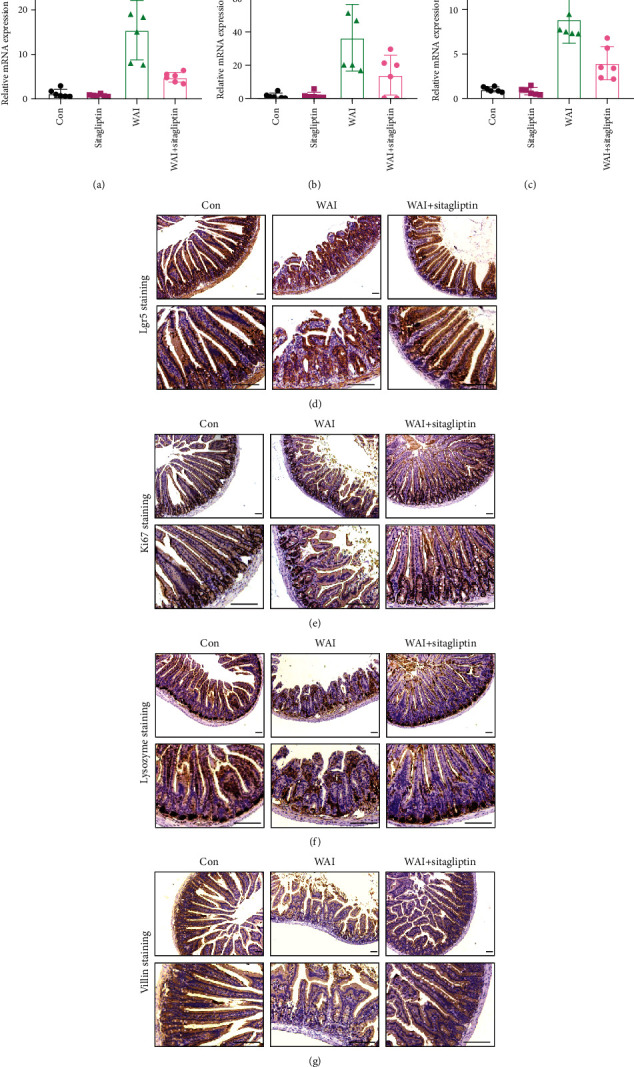
Sitagliptin inhibits intestinal inflammation and promotes the regeneration of intestinal epithelial cells in irradiated mice. (a) mRNA level of TNF*α* in small intestinal tissues, *n* = 6. (b) mRNA level of IL-6 in small intestinal tissues, *n* = 6. (c) mRNA level of IL-1*β* in small intestinal tissues. (d–g) IHC staining of Lgr5^+^, Ki67^+^, Lysozyme^+^, and Villin^+^ cells in small intestinal tissues. Scale bars, 100 *μ*m.

In the process of radiation-induced intestinal injury, various proinflammatory cytokines were involved. Proinflammatory cytokines including TNF-*α*, IL-6, and IL-1*β* have been reported to play a critical role in this process [19, 20]. Hence, we conducted qRT-PCR to detect the effects of Sitagliptin on the production of *TNFα*, *IL-6*, and *IL-1β* in intestinal tissues. We found that the proinflammatory cytokines (*TNFα*, *IL-6*, and *IL-1β*) in the intestinal tissues of irradiated mice were significantly upregulated compared with the control group. Administration with Sitagliptin could attenuate the elevated proinflammatory cytokines in the intestinal tissues of irradiated mice (Figures 2(a)–2(c)). Intestinal epithelial regeneration relies on the continuous self-renewal of intestinal stem cells (ISCs) expressing leucine-rich repeat-containing G-protein-coupled receptor 5 (Lgr5), which is essential to resist luminal stress and maintain homeostasis [21]. In our study, we found that Lgr5-expressing cells were significantly reduced in the WAI group compared with the control group, while these cells were significantly recovered under the treatment with Sitagliptin in mice after WAI (Figure 2(d)). We further performed IHC staining to detect the Ki67^+^ cells, Lysozyme^+^ cells, and Villin^+^ cells to determine the effect of Sitagliptin on proliferation and differentiation of crypt cells. Consistently, the Ki67^+^ cells, Lysozyme^+^ cells, and Villin^+^ cells were markedly reduced in the WAI group compared with the control group, and Sitagliptin treatment significantly prevented these changes (Figures 2(e)–2(g)). These results indicated that Sitagliptin could inhibit intestinal inflammation and promote the regeneration of intestinal epithelial cells in irradiated mice.

### 3.3. Sitagliptin Prevents Radiation-Induced Apoptosis and DNA Damage in Intestinal Epithelial Cells

**Figure 3:**
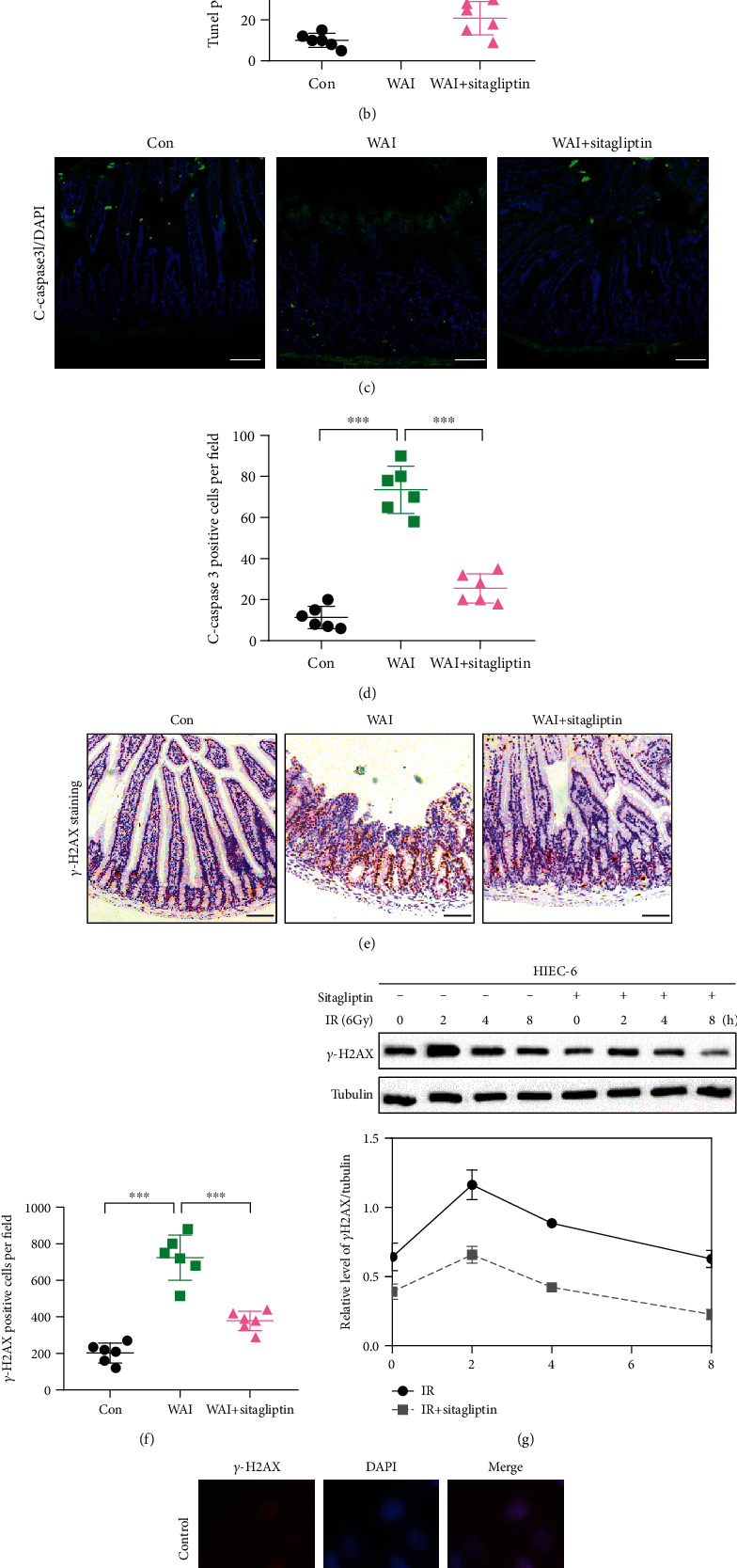
Sitagliptin prevents radiation-induced apoptosis and DNA damage in intestinal epithelial cells. (a, b) The effect of Sitagliptin on apoptosis in radiation-induced intestinal damage was assessed by TUNEL staining. Scale bars, 100 *μ*m, *n* = 6. (c, d) Representative immunofluorescence images and quantification of C-caspase3 in the small intestinal tissues. Scale bars, 100 *μ*m, *n* = 6. (e, f) Representative images and quantification of *γ*-H2AX IHC staining in small intestinal tissues. Scale bars, 100 *μ*m, *n* = 6. (g) The expression of *γ*-H2AX in HIEC-6 cells was examined by western blotting. (h) The formation of *γ*-H2AX foci was evaluated by immunofluorescence in HIEC-6 cells. Scale bars, 10 *μ*m.

To investigate the potential mechanism of the protective effect of Sitagliptin on radiation-induced intestinal injury, TUNEL assay was used to analyze the effect of Sitagliptin on apoptosis of intestinal tissue in irradiated mice. The results showed that WAI in mice contributed to more TUNEL-positive cells in intestinal crypts compared to the control group, but the administration of Sitagliptin could reduce the number of TUNEL-positive cells in the crypt of irradiated mice (Figures 3(a) and 3(b)). Additionally, we proceeded to detect the expression of cleaved-caspase-3 (C-caspase3) in the small intestine. We found that the number of activated caspase-3-positive cells in the WAI group was higher than that in the control group, while the expression of C-caspase3 was significantly inhibited in irradiated mice treated with Sitagliptin. These data suggested that treatment with Sitagliptin could reduce apoptosis in small intestinal tissue in WAI-treated mice (Figures 3(c) and 3(d)).

To explore the role of Sitagliptin in radiation-induced DNA damage repair, we used IHC staining to determine the expression of *γ*-H2AX, a biomarker of DNA double-strand break (DSB) in intestinal tissue [22]. The results are shown in Figures 3(e) and 3(f). Compared with the control group, the level of *γ*-H2AX in the small intestine of mice in the WAI group was significantly stimulated. In contrast, Sitagliptin could reduce the upregulation of *γ*-H2AX in irradiated mice. In addition, we further used human intestinal epithelial cell (HIEC) to investigate the effect of Sitagliptin on radiation-induced intestinal epithelial cell injury *in vitro.* First, the CCK-8 analysis was used to determine the dose of Sitagliptin with no obvious effect on the growth of HIEC-6 cells, i.e., ≤5 *μ*M (Figure S1). Radiation exposure led to a significant decrease in cell viability compared to the control group, and Sitagliptin treatment reversed radiation-mediated inhibition of cell growth. Then, a dose of 5 *μ*M of Sitagliptin was used in the following experiments *in vitro*. Western blot analysis of the *γ*-H2AX protein level in HIEC-6 cells showed that Sitagliptin could reduce the radiation-induced increase of *γ*-H2AX expression (Figure 3(g)). Immunofluorescence analysis also revealed that the number of *γ*-H2AX foci in radiation-exposed HIEC-6 cells was significantly higher than that in control cells, and the change was attenuated by treatment with Sitagliptin (Figure 3(h)). These results indicated that Sitagliptin could alleviate the radiation-induced DNA damage.

### 3.4. Sitagliptin Upregulates the Expression and Function of NRF2 in Intestinal Epithelial Cells

Nuclear factor erythroid-2 related factor 2 (NRF2) is a core transcription factor of the antioxidant system that participated in scavenging reactive oxygen species and protect cells from DNA damage and apoptosis after irradiation [23]. We found the level of MDA, one of the oxidative stress indexes, was significantly increased in the intestine after WAI. On the contrary, Sitagliptin treatment effectively inhibited the production of MDA in the small intestine induced by WAI (Figure S2). Thus, we asked whether the NRF2 signal is involved in Sitagliptin-induced radioprotective effects against intestinal injury in mice.

**Figure 4:**
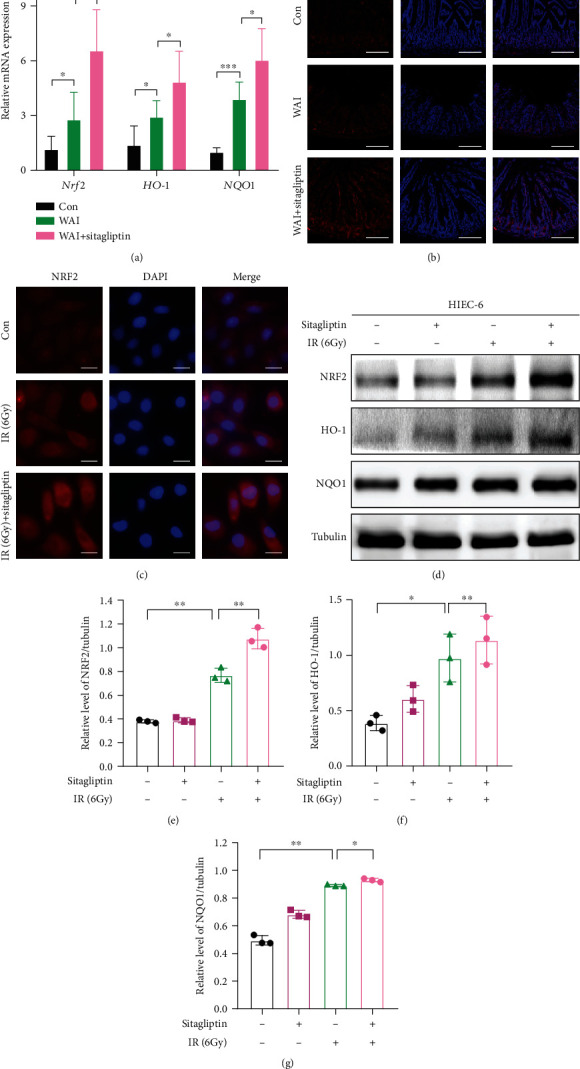
Sitagliptin upregulates the expression and function of NRF2 in intestinal epithelial cells. (a) mRNA levels of Nrf2, HO-1, and NQO1 in small intestinal tissues. (b) Immunofluorescence of NRF2, HO-1, and NQO1 in small intestinal tissues. Scale bars, 100 *μ*m. (c) Immunofluorescence of NRF2, HO-1, and NQO1 in HIEC-6 cells. Scale bars, 10 *μ*m. (d) Western blot analysis of NRF2, HO-1, and NQO1 in HIEC-6 cells. (e–g) Quantitative analysis of NRF2, HO-1, and NQO1 in HIEC-6 cells.

We measured the expression of *Nrf2* and its downstream gene *HO-1* and *NQO1* in small intestinal tissues of mice by qRT-PCR. Our results revealed that radiation exposure increased the level of *Nrf2*, *HO-1*, and *NQO1* in small intestinal tissues and these upregulations were further stimulated by treatment with Sitagliptin (Figure 4(a)). IF analysis also showed that Sitagliptin promoted the upregulation of NRF2 level in radiation-induced small intestine tissue (Figure 4(b)). In addition, we further explored the effect of Sitagliptin on the NRF2-antioxidant axis *in vitro*. Immunofluorescence analysis showed that Sitagliptin enhanced the protein expression of NRF2 in HIEC-6 cells after radiation exposure (Figure 4(c)). In addition, western blot analysis showed that the expression levels of NRF2 and its downstream antioxidant proteins HO-1 and NQO1 were upregulated by IR and further upregulated by Sitagliptin treatment (Figures 4(d)–4(g)). Taken together, our data indicated that treatment with Sitagliptin might alleviate radiation-induced damage by enhancing the NRF2-mediated antioxidant response intestinal epithelial cells.

### 3.5. Sitagliptin Attenuates Radiation-Induced Activation of NLRP3 Inflammasome in Intestinal Epithelial Cells

**Figure 5:**
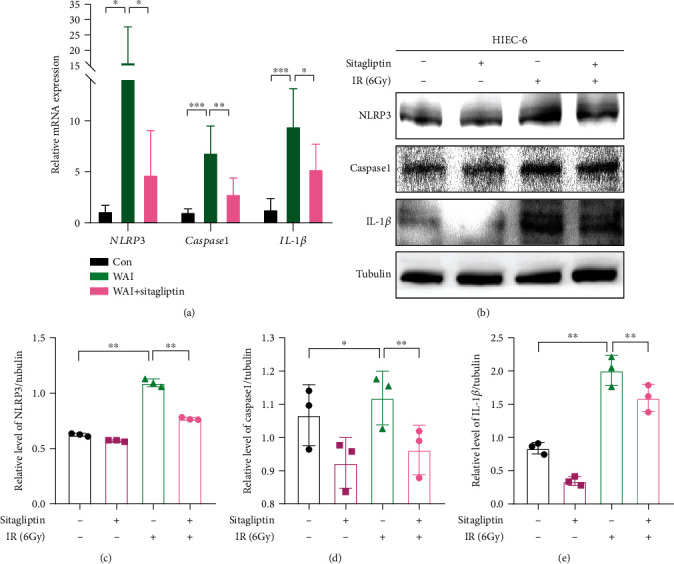
Effect of Sitagliptin on the NLRP3 inflammasome in intestinal epithelial cells. (a) mRNA levels of NLRP3, caspase-1, and IL-1*β* in small intestinal tissues. (b) Western blot assay of the protein levels of NLRP3, caspase-1, and IL-1*β* in HIEC-6 cells. (c–e) Quantitative analysis of the expression levels of NLRP3, caspase-1, and IL-1*β* normalized to Tubulin in HIEC-6 cells.

NLRP3 inflammasome is a multiprotein complex whose overactivation can contribute to the development of many inflammation-related diseases [24, 25]. In our study, we also evaluated the expression of NLRP3 inflammasome in the intestinal tissues of mice. Radiotherapy activated the mRNA level of *NLRP3*, *caspase-1*, and *IL-1β* in intestinal tissue treatment with Sitagliptin inhibiting the upregulations of them in irradiated mice (Figure 5(a)). Moreover, further *in vitro* experiments kept the results consistent. The expressions of NLRP3, caspase-1, and IL-1*β* in the irradiated group were remarkably increased, while Sitagliptin administration could strongly reduce the above protein levels in HIEC-6 cells (Figures 5(b)–5(e)). Therefore, Sitagliptin was able to attenuate radiation-induced activation of NLRP3 inflammasome in intestinal epithelial cells.

### 3.6. Sitagliptin Sensitizes Colorectal Cancer to Radiotherapy in an Animal Model

**Figure 6:**
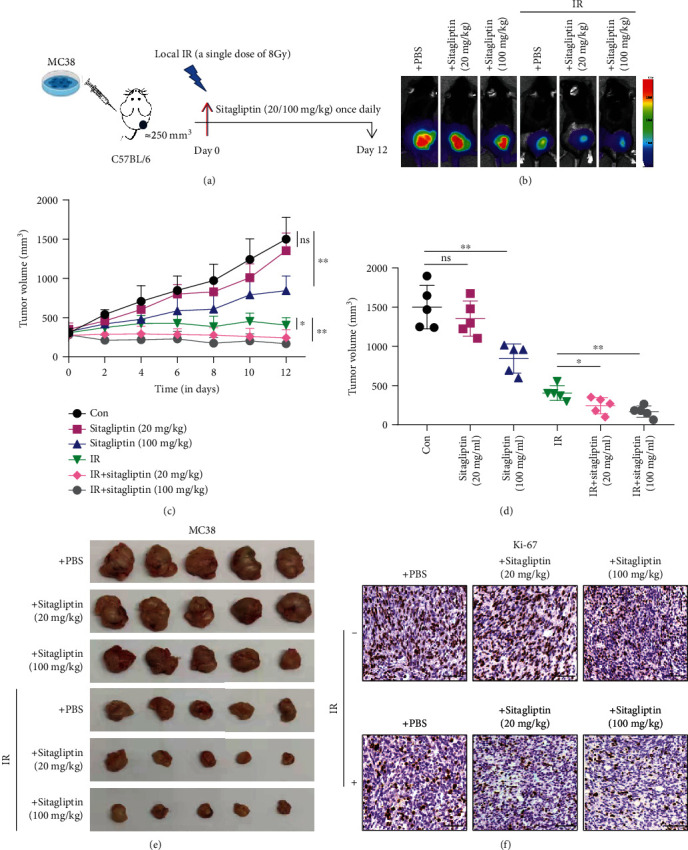
Sitagliptin sensitizes colorectal cancer to radiotherapy in an animal model. (a) Operational diagram of the animal experiment. (b) A representative image of in vivo imaging. (c) Tumor growth curve in each group, *n* = 5. (d) Tumor volume at the end point shown for each group with five mice per group, *n* = 5. (e) Representative tumor of each group. (f) Tumor proliferating cells determined by Ki-67 IHC staining. Scale bars, 100 *μ*m.

To further investigate the potential clinical application value of Sitagliptin for radiotherapy, we further examined the effect of Sitagliptin on tumor growth *in vivo*. We generated subcutaneous tumors by injecting MC38 cells (1 × 10^6^ cells/mouse) into C57BL/6 mice. Mice bearing established subcutaneous tumors (tumor volume approximately 250 mm^3^) were treated with various treatments as shown in Figure 6(a). In our tumor experiment, we found that 8 Gy local IR alone partially inhibited the growth of MC38 cell tumor, while low dose of 20 mg/kg Sitagliptin alone had no significant inhibitory effect on tumor growth, and a high dose of 100 mg/kg Sitagliptin slightly inhibited the tumor growth. The combination of local IR with low or high concentrations of Sitagliptin significantly enhanced tumor growth inhibition compared to local IR alone (Figures 6(b)–6(e)). IHC analysis of the level of the cell proliferation marker Ki-67 showed that the local IR and high-dose Sitagliptin could reduce the expression of Ki-67 in tumors, and the lowest expression was observed in the combined treatment group (Figure 6(f)). Overall, the present results support that Sitagliptin could sensitize MC38 tumors to IR.

### 3.7. Sitagliptin Restores Gut Bacterial Composition Pattern Prejudiced by WAI

**Figure 7:**
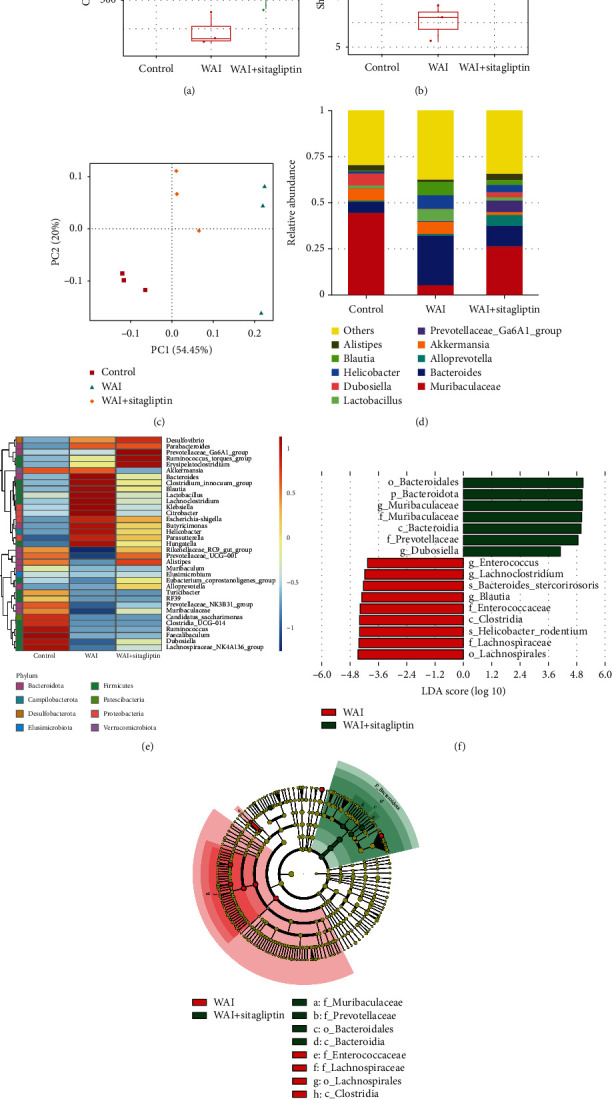
Effects of Sitagliptin on the gut bacterial composition pattern. The feces in different groups were collected 3.5 days after WAI for 16S rRNA sequencing, *n* = 3. (a, b) Chao1 and Shannon indexes. (c) PCoA based on weighted unifrac. (d) The relative abundance of the top 10 intestinal bacteria at the genus level. (e) The differences of intestinal bacteria at the genus level showed in heat map. (f) Linear discriminant analysis (LDA) effect size (LEfSe) analysis between the WAI and WAI+Sitagliptin groups. (g) LEfSe cladogram between the WAI and WAI+Sitagliptin groups.

Some previous studies have reported that gut microbiota could affect the host's radioresistance [26, 27]; we therefore analyzed the effects of Sitagliptin on gut bacterial composition following the WAI in mice. As shown in Figures 7(a) and 7(b), the Chao1 index and the Shannon index were observably decreased after WAI, while Sitagliptin treatment reduced the alterations. Moreover, the principal coordinate analysis (PCoA) demonstrated that the gut bacterial structures were significantly changed after WAI and Sitagliptin administration (Figure 7(c)). More specifically, at the genus level, the relative abundance of *Bacteroides*, *Blautia*, *Helicobacter*, and *Lactobacillus* was increased, and the relative abundance of *Alistipes* and *Dubosiella* was decreased in the WAI group, whereas Sitagliptin treatment restored the changes of bacteria abundances caused by WAI (Figures 7(d) and 7(e)). Linear discriminant analysis (LDA) and LDA effect size (LEfSe) analysis were further applied to identify differences in taxa abundance between the WAI and WAI+Sitagliptin groups at the genus level. We observed that the genus *Enterococcus* and *Lachnoclostridium* were significantly more abundant in the WAI group compared with the WAI+Sitagliptin group, while the genus *Muribaculaceae* and *Dubosiella* were enriched in the WAI+Sitagliptin group (Figures 7(f) and 7(g)). These results indicated that Sitagliptin might play a radioprotective role by restoring the gut bacterial composition pattern impaired by WAI.

## 4. Discussion

Radiotherapy is a common strategy for radical or palliative treatment of tumors. High doses of ionizing radiation to abdominal and pelvic tumors can disrupt the intestinal epithelial barrier, followed by mucosal inflammation and bacterial translocation, resulting in acute gastrointestinal radiation syndrome (AGS) characterized by nausea, diarrhea, electrolyte disorders, bacteremia, and septicemia [28]. Severe intestinal injury can lead to treatment termination or even death, which greatly limits the radiation dose and affects the therapeutic effect in clinical practice.

In recent years, various models such as mouse model, rat model, minipig model, and organoid model have been widely used in the study of radiation-induced intestinal injury [29–32]. In this study, C57BL/6 mice were irradiated with 12.5 Gy of whole abdomen, and obvious hair changes appeared in the irradiation field about 20 days later. Mice suffered weight loss, diarrhea, reduced food intake, and even death after radiotherapy. Intestinal tissues of mice were examined at 3.5 d, and we found that radiotherapy caused severe intestinal hyperemia and hemorrhagic changes and reduced intestinal contents. HE staining analysis showed that radiotherapy could lead to the destruction of intestinal villus-crypt structure, further confirming that we successfully constructed a radiation-induced intestinal injury model *in vivo*. The *in vitro* model of radiation-induced intestinal injury constructed by intestinal epithelial cells was also used in our study, to further evaluate the efficacy and mechanism of the drug.

Sitagliptin, a DDP-4 inhibitor was extensively used in the treatment of type 2 diabetes. Accumulated evidence suggests that Sitagliptin not only regulates blood glucose but also has pleiotropic effects of anti-inflammatory, antioxidant, and antiapoptotic. Sitagliptin has been reported to have therapeutic effects on radiation-induced hematopoietic damage [33]. In our study, we found that once-daily treatment with Sitagliptin starting 1 hour before radiotherapy could protect the mice from death and weight loss induced by WAI. Consistent with what has been reported in mice treated with TBI, Sitagliptin could alleviate the elevated level of proinflammatory cytokines (*TNFα*, *IL-6*, and *IL-1β*) in mice treated with WAI. Moreover, Sitagliptin could increase the survival of Lgr5^+^ cells, Ki67^+^ cells, Lysozyme^+^ cells, and Villin^+^ cells in the small intestines of irradiated mice to maintain the intestinal homeostasis and promote the regeneration of ISCs. In addition, administration of Sitagliptin significantly reduced the number of TUNEL-positive cells and lowered the levels of activated caspase-3 and phosphorylated histone H2AX. Overall, these results suggested that Sitagliptin can prevent radiation-induced intestinal injury by inhibiting intestinal inflammation, promoting ISC regeneration, and attenuating radiation-induced apoptosis and DNA damage in intestinal epithelial cells.

We further explored the therapeutic targets and molecular mechanisms of Sitagliptin in alleviating radiation-induced intestinal injury. It is well known that NRF2 is a core transcription factor of the antioxidant response system. Once subjected to the oxidative stress, NRF2 can be transferred to the nucleus and bind to the antioxidant response element (ARE) to induce the transcription of target genes, thereby activating downstream antioxidant proteins, such as hemeoxygenase-1 (HO-1) and NAD(P) H quinone oxidoreductase-1 (NQO1), to defend against oxidative stress [34]. Sitagliptin has been reported to relieve acute pancreatitis-associated acute lung injury and intestinal inflammation by activating NRF2 [7, 35]. Our results showed that administration with Sitagliptin significantly reversed the increase of MDA in intestinal tissues of irradiated mice. Meanwhile, treatment with Sitagliptin further promoted the activation of the NRF2-HO-1/NQO1 signaling pathway in both irradiated intestinal tissues and HIEC-6 cells. However, although studies have shown that DDP4 is negatively correlated with NRF2 and DDP4 can be upregulated after NRF2 knockout [7], the specific molecular mechanism of Sitagliptin regulating NRF2 activation still needs to be further studied.

Many different stimuli can trigger uncontrolled activation of NLRP3 inflammasome, thus promoting the progression of systemic inflammation and adaptive immune responses. Upregulated NLRP3 promotes caspase-1-mediated inflammatory cascade and induces the expression of downstream proinflammatory factors, such as IL-1*β*, IL-6, and TNF-*α* [24, 36]. It has been reported that NLRP3 inflammasome-mediated pyroptosis is involved in exacerbating radiation-induced intestinal injury and radiation-induced cardiovascular injury [37, 38]. Here, we demonstrated that NLRP3, caspase-1, and IL-1*β* were activated in a radiation-induced mouse model of intestinal injury and cell culture model. Sitagliptin could inhibit radiation-induced NLRP3 inflammasome activation and reduced the secretion of downstream proinflammatory cytokines. These results suggest that Sitagliptin may alleviate radiation-induced intestinal injury by inhibiting the activation of NLRP3 inflammasome. A close crosstalk between the antioxidant stress pathway and the NLRP3 inflammasome has been widely reported [39–41]. Here, Sitagliptin could promote NRF2 expression and inhibits NLRP3 inflammasome activation. However, it remains to be determined whether Sitagliptin inhibited NLRP3 inflammasome relying on the activation of NRF2.

In addition, we also investigated the effects of Sitagliptin on gut microbiota after radiation. Previous studies have shown that gut microbiome disturbance plays a key role in the development of radiation-induced intestinal injury [42]. We performed 16S rRNA sequencing on mouse feces in each group and found that abdominal irradiation could change the composition of intestinal flora with decreased abundance and diversity, suggesting an increase in intestinal inflammation susceptibility. However, oral administration of Sitagliptin significantly alleviated intestinal microbial disorder and restored intestinal microbial abundance in irradiated mice, suggesting that the regulation of gut microbes is also one of the mechanisms by which Sitagliptin ameliorates radiation-induced intestinal injury.

As an inhibitor of DDP4, the efficacy of Sitagliptin in tumor control is controversial. A retrospective analysis showed that DDP-4 inhibitors appeared to improve survival in patients with prostate cancer, but not in those with pancreatic and breast cancer [43]. It has also been reported that DPP-4i can promote NRF2 activation and accelerate tumor metastasis [44]. Intriguingly, our research suggests that besides the radiation protection, Sitagliptin can also sensitize colorectal cancer to radiotherapy in animal models. The results indicated that the combination of local IR and Sitagliptin, at a low concentration or high concentration, both had a greater effect on reducing MC38 tumor size than treatment with local IR alone. Similarly, previous studies have shown that Sitagliptin combined with chemotherapy, targeted therapy, or immunotherapy makes better antitumor efficacy. Sitagliptin enhances chemotherapy response of ovarian cancer by modulating the intracellular transmission pathways [45]. DPP4 has also been recently identified as a cancer stemness-related protein whose inhibition can rescue tyrosine kinase inhibitor resistance in renal cell carcinoma [46]. Sitagliptin could activate NK and T cell chemotaxis through preservation of the CXCR3-CXCL10 axis and suppress tumor angiogenesis in hepatocellular carcinoma, thereby inhibiting tumor growth and improving the immunotherapy effect [47]. However, the mechanism of Sitagliptin enhancing radiosensitivity in colorectal cancer needs to be further explained.

## 5. Conclusion

In summary, our results demonstrated that Sitagliptin might be a promising candidate for the therapeutics of radiation-induced intestinal injury by regulating oxidative damage, inflammation, and gut microbiota disorder.

## Data Availability

All data are included in this manuscript to support the findings of this study.

## Supplementary Materials

Supplementary Materials Figure S1: Sitagliptin inhibits IR-induced suppression of viability, oxidative stress, and DNA damage of HIEC-6 cells. (A, B) Cell viability was measured using the CCK-8 assay. Figure S2: Sitagliptin reversed the increase of MDA in irradiated mice. Table S1: the sequences of primers used for experiments in this study.
