# Supplementary material for: Sitagliptin Alleviates Radiation-Induced Intestinal Injury by Activating NRF2-Antioxidant Axis, Mitigating NLRP3 Inf--lammasome Activation, and Reversing Gut Microbiota Disorder
Source: Oxid Med Cell Longev. 2022 May 17;2022:2586305. doi: 10.1155/2022/2586305 (PMC9129991; doi:10.1155/2022/2586305)
Supplement: Supplementary Materials — Figure S1: Sitagliptin inhibits IR-induced suppression of viability, oxidative stress, and DNA damage of HIEC-6 cells. (A, B) Cell viability was measured using the CCK-8 assay. Figure S2: Sitagliptin reversed the increase of MDA in irradiated mice. Table S1: the sequences of primers used for experiments in this study. [file 2586305.f1.docx]

**Figure S1.** Sitagliptin inhibits IR-induced suppression of viability, oxidative stress and DNA damage of HIEC-6 cells.


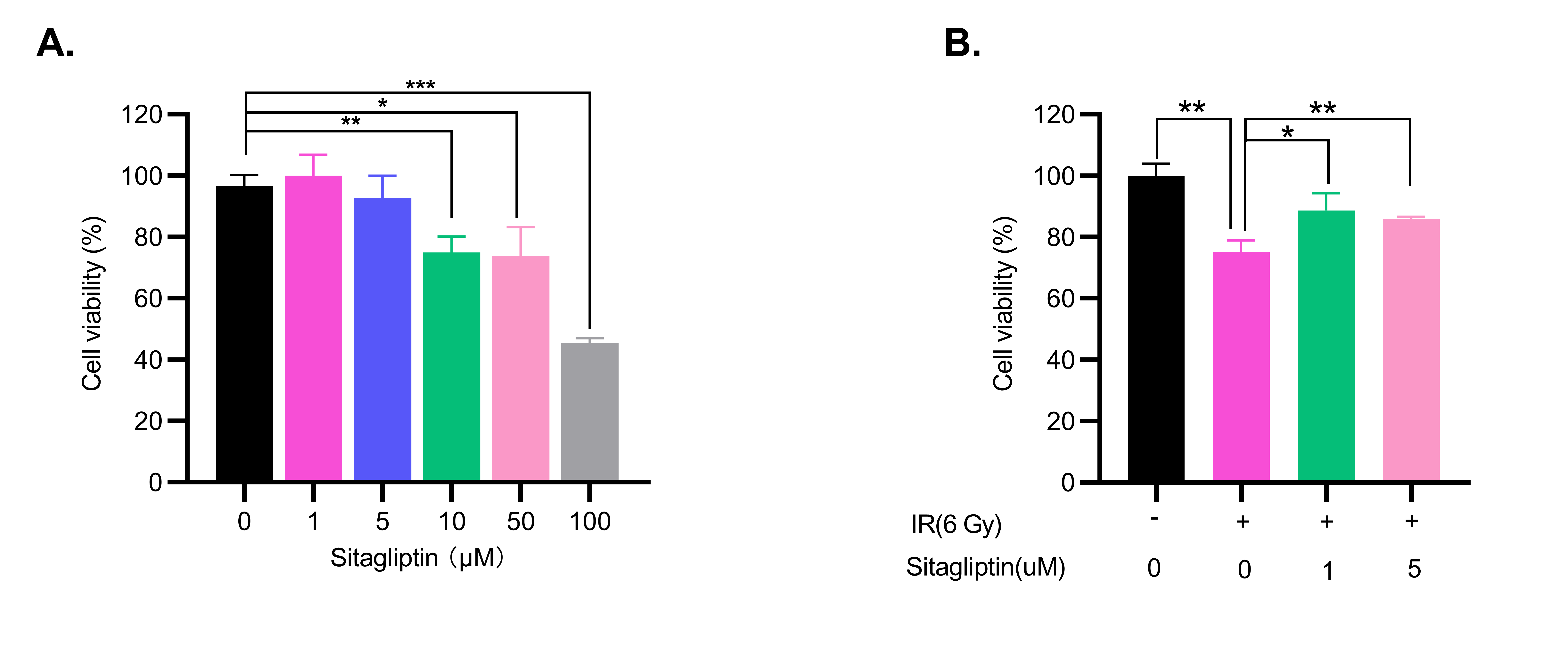


(A, B) Cell viability was measured using the CCK-8 assay.

**Figure S2.** Sitagliptin reversed the increase of MDA in irradiated mice.


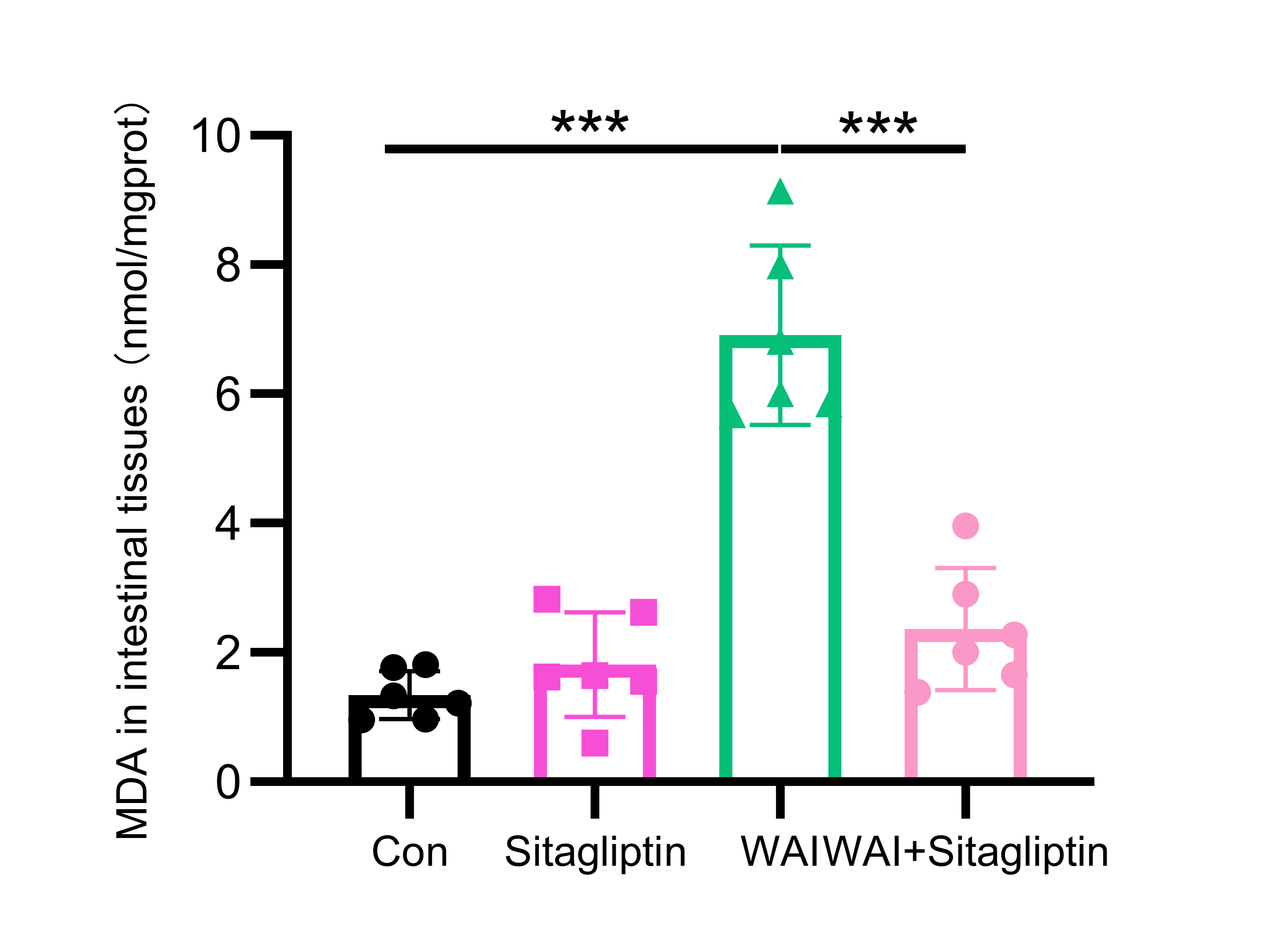


| **Table S1.** The sequences of primers used for experiments in this study. | | |
| --- | --- | --- |
| Gene | Forward primer (5′–3′) | Reverse primer (5′–3′) |
| β-actin | GGCTGTATTCCCCTCCATCG | CCAGTTGGTAACAATGCCATGT |
| TNF-α | CCCTCACACTCAGATCATCTTCT | GCTACGACGTGGGCTACAG |
| IL-6 | TAGTCCTTCCTACCCCAATTTCC | TTGGTCCTTAGCCACTCCTTC |
| Nrf2 | TAGATGACCATGAGTCGCTTGC | GCCAAACTTGCTCCATGTCC |
| HO-1 | AAGCCGAGAATGCTGAGTTCA | GCCGTGTAGATATGGTACAAGGA |
| NQO1 | TGGCCGAACACAAGAAGCTG | GCTACGAGCACTCTCTCAAACC |
| NLRP3 | ATTACCCGCCCGAGAAAGG | TCGCAGCAAAGATCCACACAG |
| Caspase1 | ACAAGGCACGGGACCTATG | TCCCAGTCAGTCCTGGAAATG |
| IL-1β | GCAACTGTTCCTGAACTCAACT | ATCTTTTGGGGTCCGTCAACT |
